# Putative Zinc Finger Protein Binding Sites Are Over-Represented in the Boundaries of Methylation-Resistant CpG Islands in the Human Genome

**DOI:** 10.1371/journal.pone.0001184

**Published:** 2007-11-21

**Authors:** Shicai Fan, Fang Fang, Xuegong Zhang, Michael Q. Zhang

**Affiliations:** 1 MOE Key Laboratory of Bioinformatics and Bioinformatics Division, TNLIST, Department of Automation, Tsinghua University, Beijing 100084, China; 2 Cold Spring Harbor Laboratory, Cold Spring Harbor, New York, United States of America; 3 School of Automation Engineering, University of Electronic Science and Technology of China, Chengdu, China; Deutsches Krebsforschungszentrum, Germany

## Abstract

**Background:**

Majority of CpG dinucleotides in mammalian genomes tend to undergo DNA methylation, but most CpG islands are resistant to such epigenetic modification. Understanding about mechanisms that may lead to the methylation resistance of CpG islands is still very poor.

**Methodology/Principal Findings:**

Using the genome-scale *in vivo* DNA methylation data from human brain, we investigated the flanking sequence features of methylation-resistant CpG islands, and discovered that there are several over-represented putative Transcription Factor Binding Sites (TFBSs) in methylation-resistant CpG islands, and a specific group of zinc finger protein binding sites are over-represented in boundary regions (∼400 bp) flanking such CpG islands. About 77% of the over-represented putative TFBSs are conserved among human, mouse and rat. We also observed the enrichment of 4 histone methylations in methylation-resistant CpG islands or their boundaries.

**Conclusions/Significance:**

Our results suggest a possible mechanism that certain putative zinc finger protein binding sites over-represented in the boundary regions of the methylation-resistant CpG islands may block the spreading of methylation into these islands, and those TFBSs over-represented within the islands may both reinforce the methylation blocking and promote transcription. Some histone modifications may also enhance the immunity of the CpG islands against DNA methylation by augmenting these TFs' binding. We speculate that the dynamical equilibrium between methylation spreading and blocking is likely to be responsible for the establishment and maintenance of the relatively stable DNA methylation pattern in human somatic cells.

## Introduction

DNA methylation is one of the most important epigenetic modifications. In mammalian genomes, DNA methylation occurs at the cytosine residue in the context 5′-CG-3′ (CpG dinucleotide) by virtue of DNA methyltransferases [1]. It has been reported that DNA methylation plays many important functional roles such as X-chromosome inactivation [2], [3], the establishment and maintenance of tissue-specific gene expression profiles [4]–[6] as well as developmental programming regulation [7], [8].

In human somatic cells, about 70–80% of CpG dinucleotides undergo methylation [9]. Unmethylated CpG dinucleotides largely reside in genomic regions called CpG islands (CGIs) [1]. A CGI is a region where the CpGs are more dense than the genome average, commonly defined as a region with length of 200 bp or longer in which the G+C content is no less than 50% and the ratio of observed/expected CpGs is larger than 0.6 [10]. Although some CGIs could be methylated in the imprinted regions [4] or in the inactive X-chromosome [3], [11], [12], most CGIs are generally resistant to DNA methylation [13]. But so far it is still poorly understood what mechanisms may lead to the methylation-resistance of these CGIs.

A few biological experiments focusing on some specific genes have indicated that certain *cis*-acting elements could act as boundaries to protect CGIs from methylation (by binding to the corresponding TFs), such as Sp1 elements in mouse *aprt* gene [14], [15], the (ATAAA)_n_ repeated sequences in human *GSTP1* gene [16], and Sp1 and CTCF elements in *BRCA1* gene [17]. These elements are not universal, and it has been observed that the deletion of Sp1 in mouse *aprt* gene would not cause aberrant methylation of CGIs [18]. Therefore there must be some other *cis*-acting elements performing similar boundary roles and/or some other mechanisms that may help to resist methylation. In addition, there is little knowledge about location and length, if exist, of the protective boundaries around unmethylated CGIs.

In recent years, there emerged some computational works on the prediction of the methylation status of CpG dinucleotides [19], CGIs [20], [21], and CGI fragments [22], [23] based solely on DNA sequence features . They could reach prediction accuracies around 80%, which partially confirms people's speculation that there may exit sequence propensity for genomic DNA methylation.

Inspired by these success, we conducted a further investigation on the potential flanking sequence features of methylation-resistant CGIs (referred to as un-methylated or U-CGIs for simplicity) with a genome-scale methylation profiling dataset from human brain [24]. We found that certain zinc finger protein binding sites, including Sp1 and CTCF sites, are over-represented in the boundary sequences of U-CGIs, and the core region of such boundaries appears to extend ∼400 bp upstream or downstream from the island. There are also some over-represented *cis*-acting elements within U-CGIs, which may have dual functions of blocking DNA methylation spreading and promoting transcription. These results were validated to a certain degree on two other independent large-scale datasets. The study on a recent genome-scale histone modification dataset showed the enrichment of the four histone methylations in the U-CGIs or their boundaries, which implies that many of the TFs indeed bind to some of their putative binding sites. Based on our observations on these genome-scale data, we believe that the dynamical equilibrium hypothesis proposed for mouse [25] could be generalized to human: during the *de novo* methylation process in the genome, repetitive sequences (such as *Alu* sequences) act as DNA methylation center. As methylation spreads towards a CGI, several *cis*-acting elements in the boundaries of the CGI can recruit specific DNA binding proteins, such as Sp1 and CTCF, as battlefronts to prevent DNA methylation from encroaching into the CGI, and additional *cis*-elements within the U-CGI regions can further help reinforcing the methylation blocking activities. And these enriched histone methylations will also enhance the immunity of U-CGIs from methylation by augmenting the TFs' binding. These opposing (spreading and blocking) activities would come to a dynamical equilibrium under a given physiological condition of the cells to establish the observed genomic methylation patterns.

## Results

We got 304 U-CGIs and their corresponding flanking sequences from the methylation-resistant domains of Rollins *et al*'s genome-scale methylation data from human brain (see Materials and methods for details). Another 210 methylation-prone CGIs (referred to as methylated CGIs or M-CGIs) and their flanking sequences were extracted from the methylation-prone domains of the same data set to serve as the background control samples. We used these data to explore the *cis*-element features of U-CGIs and their flanking sequences that are distinct from those of M-CGIs and their flanking sequences.

**Figure 1:**
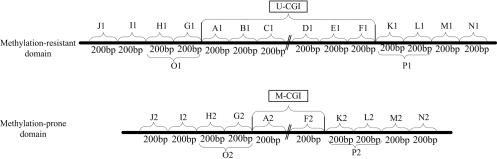
Sketch of the division strategies. We split U&M-CGIs into several fragments with equal width of 200 bp. As the median length of U-CGIs are much longer than M-CGIs, there are 6 fragments of U-CGIs (A1, B1, C1, D1, E1, F1) whereas there are only 2 fragments of M-CGIs (A2 and F2), and such fragments could cover most of the sequences of U&M-CGIs. Applying the same division strategy to the flanking sequences corresponding to both U&M-CGIs, we extended 800 bp upstream and downstream of CGIs, and got fragments of J1, I1, H1, G1, K1, L1, M1, N1, and J2, I2, H2, G2, K2, L2, M2, N2 respectively. We further define 400 bp upstream and downstream of U-CGIs (O1, P1) as the boundary sequences since there are few significant TFBSs outside such regions.

**Figure 2:**
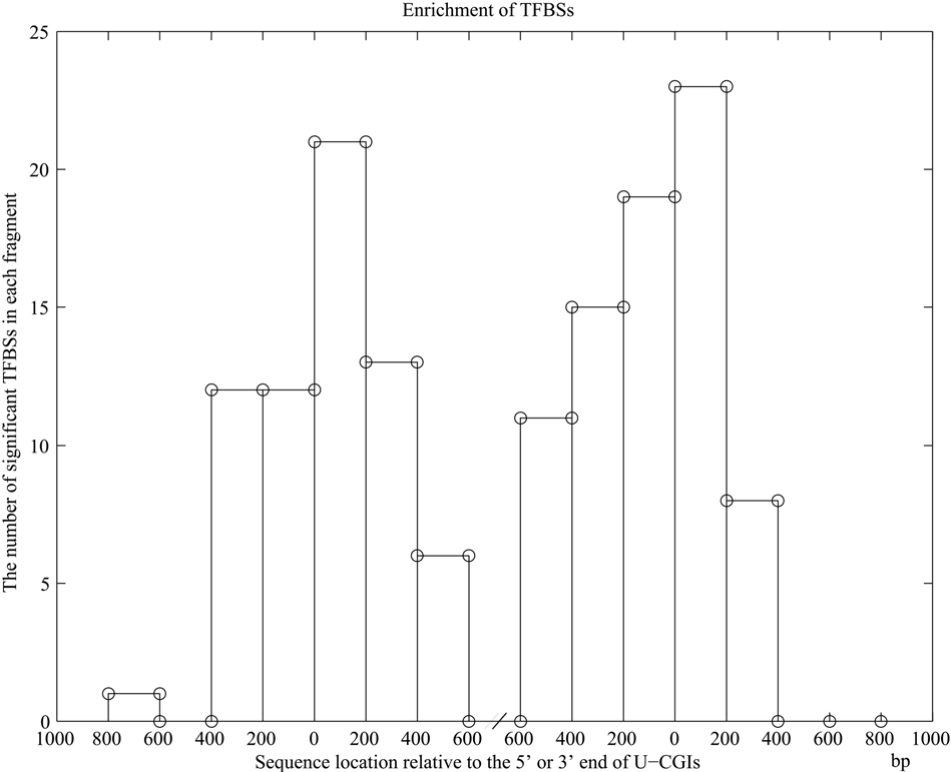
The number of significantly over-represented TFBSs in each fragment of U-CGIs and their flanking sequences. The *x*-axis indicates the location of fragment relative to the 5′ or 3′ end of U-CGIs. Fragments insides the two ‘0’s correspond to A1, B1, C1, D1, E1 and F1 of Figure 1, and fragments that are in the 800 bp upstream and downstream of U-CGIs represent J1, I1, H1, G1 and K1, L1, M1, N1 of Figure 1 respectively. The *y*-axis is the number of significantly enriched TFBSs in each fragment. We applied the Bonferroni-adjusted *p*-value cutoff 0.01 here. The sequences that are 400 bp upstream and downstream of U-CGIs are defined as the boundary sequences.

For studying the sequence features, we split U-CGIs, M-CGIs and their corresponding flanking sequences into several 200 bp-long fragments (motivated by the average inter-nucleosome distance) as illustrated in Figure 1, and all samples were aligned by the two ends of the CpG islands (see Materials and Methods for details). Then MOTIFCLASS [26] was applied to investigate the Transcription Factor Binding Site (TFBS) frequencies both in the fragments of U-CGIs and their flanking sequences. TFBSs used here are from TRANSFAC 9.4 [27]. We used a two-step hypothesis test for this study (see Materials and Methods for details), and obtained all the significantly enriched TFBSs (Bonferroni-adjusted *p*-value cutoff 0.01) for each fragment of U-CGIs and flanking sequences. The enrichment of TFBSs is shown in Figure 2. In the figure, the region between the two ‘0’s on the *x*-axis represents the U-CGI region, the regions outside the two ‘0’s are the flanking regions, and the *y*-axis is the number of significant TFBSs in each fragment. From Figure 2, one can see that there are many enriched TFBSs in and around the U-CGIs, and they reach “plateaus” around the two ends of U-CGIs. These regions extend ∼400 bp upstream and downstream from the U-CGIs.

To study whether these results are sensitive to the choice of *p*-value cutoff, we also experimented with other cutoffs. The number and distribution of significant TFBSs under *p*-value cutoffs of 0.05 and 0.001 (Bonferroni-adjusted) are shown in Figures S1 and S2 in Supplementary Materials. One can see that although the specific number of significant TFBSs in each fragment may vary with the different cutoffs, the fact that there are some common enriched TFBSs in U-CGIs and in their nearby flanking sequences remains to be true. One may further define the 400 bp upstream and downstream of U-CGIs as the boundary sequences (shown as O1 and P1 regions in Figure 1), as such flanking regions appear to be more robustly containing the enriched TFBSs.

### Over-represented TFBSs in methylation-resistant CGIs

For extracting the specific TFBSs that are enriched in U-CGIs, we took TFBSs that are simultaneously significantly enriched in more than three U-CGI fragments (i.e., half of the counted fragments) as over-represented TFBSs in U-CGIs. Ten TFBSs satisfy this criterion and they are listed in Table 1 (their corresponding motif logos are given in Table S1 in Supplementary Materials). The *p*-values of these TFBSs by the two-step test were also listed in Table 1. We looked up the tissue expression pattern of the transcription factors (TFs) corresponding to these TFBSs in the database TissueDistributionDBs (http://genius.embnet.dkfz-heidelberg.de/menu/tissue_db/). Of the 10 over-represented TFBSs, 8 have been reported to be expressed in the brain (shown in the 2nd column of Table 1).

**Table 1:** Over-represented TFBSs and other novel motifs in U-CGI fragments.

Over-represented TFBS	Expressed in human brain*	A1	B1	C1	D1	E1	F1
V$KROX_Q6	Y	**4.23e-07**	**1.75e-14**	**1.35e-17**	**4.96e-20**	**2.04e-18**	**6.24e-11**
		**0.000**	**0.000**	**0.000**	**0.000**	**0.000**	**0.000**
V$SP1_01	Y	**6.65e-09**	**1.33e-11**	**1.78e-10**	**2.66e-13**	**1.27e-10**	**4.07e-10**
		**0.000**	**0.001**	**0.003**	**0.000**	**0.000**	**0.000**
V$HEN1_01	Y	**1.81e-10**	**2.62e-08**	**1.78e-10**	**1.35e-07**	**5.41e-07**	**1.12e-08**
		**0.000**	**0.001**	**0.006**	**0.001**	**0.003**	**0.002**
V$CACBINDINGPROTEIN_Q6	N	**2.71e-05**	**1.54e-06**	**1.83e-05**	**2.09e-05**	**1.67e-12**	**8.08e-08**
		**0.000**	**0.000**	**0.000**	**0.003**	**0.000**	**0.000**
V$PTF1BETA_Q6	N	**5.90e-13**	**2.23e-08**	3.25e-04	**5.32e-12**	**7.41e-08**	**3.59e-16**
		**0.000**	**0.000**	0.091	**0.000**	**0.005**	**0.000**
V$AP4_01	Y	**2.92e-12**	**2.73e-09**	2.80e-06	1.71e-05	**3.95e-08**	**7.75e-13**
		**0.000**	**0.002**	0.014	0.012	**0.001**	**0.000**
V$DR1_Q3	Y	**1.14e-14**	**1.72e-08**	7.77e-05	4.93e-05	**1.92e-07**	**1.29e-11**
		**0.000**	**0.000**	0.022	0.013	**0.000**	**0.000**
V$ELK1_01	Y	**4.20e-12**	**4.83e-10**	1.78e-05	**3.89e-08**	6.21e-06	**1.12e-11**
		**0.000**	**0.000**	0.033	**0.001**	0.015	**0.000**
V$PPAR_DR1_Q2	Y	**7.82e-09**	**6.29e-09**	1.49e-03	7.81e-05	**2.07e-07**	**1.40e-10**
		**0.000**	**0.000**	0.218	0.087	**0.009**	**0.003**
V$HEB_Q6	Y	**2.90e-06**	**2.62e-08**	2.01e-04	**2.11e-05**	3.21e-04	**8.13e-08**
		**0.000**	**0.001**	0.022	**0.000**	0.140	**0.000**
DME_CGI_1	-	**1.98e-08**	**1.51e-13**	**6.16e-22**	**2.70e-14**	3.12e-03	**2.55e-09**
		**0.001**	**0.000**	**0.000**	**0.000**	0.009	**0.000**
DME_CGI_2	-	**1.55e-08**	**4.51e-06**	**2.56e-20**	5.10e-04	**2.84e-8**	**3.86e-19**
		**0.000**	**0.000**	**0.000**	0.003	**0.001**	**0.000**

We use matrix entry of TRANSFAC which could be mapped uniquely to denote TFBS in the first column, and similarly in the tables hereinafter. The second column indicates whether the TFs corresponding to the over-represented TFBSs have been reported to express in human brain. The two numbers in the 3th–8th columns are the *p*-values in the two-step hypothesis test in the corresponding fragment (we use *p*_1_ and *p*_2_ to represent the upper and lower value respectively). Only the TFBS with *p*_1_ less than Bonferroni-adjusted *p*-value cutoff 0.01 and *p*_2_ less than 0.01 is regarded as a significantly enriched TFBS in the fragment, and is marked in bold. Here we regard TFBSs or novel motifs that are simultaneously significant in more than 3 U-CGI fragments as over-represented TFBSs or over-represented novel motifs in U-CGIs.

* Y: The TF corresponding to the TFBS has been reported to express in human brain; N: The TF corresponding to the TFBS has not been reported to express in human brain; -: No information about the TF corresponding to the motif.

Similarly in the tables hereinafter.

We checked the annotations of these TFs in Swiss-Prot (http://cn.expasy.org/), and found that among the 8 TFs reported to be expressed in human brain, the basic helix-loop-helix domain is shared by 3 TFs. To study the significance of this observation, we took the rest non-redundant vertebrate TFs in TRANSFAC 9.4 that are expressed in human brain as control and compared the number of these TFs that contain the basic helix-loop-helix domain (Table 2). One can see that the fraction of basic helix-loop-helix domain in the U-CGIs-over-represented TFs is marginally higher than that in the control TFs (*p*-value = 0.0544 by Fisher's Exact Test).

**Table 2:** The number of TFs with specific domains.

Domain	TFs in U-CGIs	TFs in control
Basic helix-loop-helix domain	3	9
Others	5	82

We list the number of TFs with basic helix-loop-helix domain and other domains in U-CGIs and control set.

To investigate whether there are some novel motifs over-represented in the U-CGIs, we applied the program DME-b [26] to search for new motifs and applied the two-step hypothesis test to get the over-represented ones. After eliminating motifs that were similar to over-represented TFBSs, we got two over-represented novel motifs. They are also listed at the bottom of Table 1, and their logos are listed in supplementary Table S3 together with the corresponding TRANSFAC TFBSs that are most similar to.

### Over-represented TFBSs in the boundaries of U-CGIs

In the boundary regions of U-CGIs, we took the TFBSs that are both significantly enriched in O1 segment and P1 segment as the over-represented TFBSs in the boundaries. There are 13 such TFBSs and they are listed in Table 3 with their corresponding *p*-values. Their motif logos are in the supplementary Table S2. The corresponding TFs for 10 of these TFBSs are reported as expressed in human brain according to TissueDistributionDBs (also shown in Table 3). By checking the annotation in Swiss-Prot, 7 of these 10 TFs possess zinc finger (C2H2-type) domains (Table 4). Also the rest non-redundant vertebrate TFs expressed in human brain are chosen as the control and the number of TFs with such domain in control set is shown in Table 4. One can see that the fraction of zinc finger domain in these TFs corresponding to the over-represented TFBSs in U-CGI boundaries is much higher than that in the control set (*p*-value = 8.63e-4 by Fisher's Exact Test).

**Table 3:** Over-represented TFBSs and other novel motifs in boundary sequences.

Over-represented TFBS	Expressed in human brain	O1	P1
V$MAZR_01	Y	**8.42e-09**	**3.32e-12**
		**0.000**	**0.000**
V$CTCF	Y	**2.16e-08**	**5.73e-08**
		**0.000**	**0.000**
V$ETF_Q6	N	**3.81e-08**	**2.06e-12**
		**0.000**	**0.000**
V$AP2_Q3	Y	**5.81e-08**	**3.42e-07**
		**0.000**	**0.000**
V$SPZ1_01	Y	**2.03e-07**	**2.28e-08**
		**0.002**	**0.000**
V$KROX_Q6	Y	**1.65e-06**	**2.10e-09**
		**0.000**	**0.000**
V$CACBINDINGPROTEIN_Q6	N	**3.52e-06**	**1.92e-05**
		**0.000**	**0.000**
V$NFKB_Q6	Y	**8.81e-06**	**4.68e-06**
		**0.000**	**0.000**
V$TFIII_Q6	Y	**2.04e-05**	**9.12e-08**
		**0.000**	**0.000**
V$MINI19_B	N	**2.10e-05**	**2.88e-06**
		**0.001**	**0.000**
V$GC_01	Y	**3.61e-05**	**1.55e-06**
		**0.000**	**0.000**
V$SP3_Q3	Y	**5.20e-05**	**2.93e-07**
		**0.000**	**0.000**
V$SP1_01	Y	**5.22e-05**	**1.92e-08**
		**0.000**	**0.000**
DME_Boundary_1	-	**3.80e-6**	**4.17e-8**
		**0.000**	**0.000**
DME_Boundary_2	-	**1.04e-5**	**1.08e-5**
		**0.000**	**0.000**

The second column indicates whether the TFs corresponding to the over-represented TFBSs have been reported to express in human brain. The two numbers in the 3th–4th columns are the *p*-values in the two-step hypothesis test in the corresponding fragment (we use *p*_1_ and *p*_2_ to represent the upper and lower value respectively). Only the TFBS with *p*_1_ less than Bonferroni-adjusted *p*-value cutoff 0.01 and *p*_2_ less than 0.01 is regarded as a significant TFBS in the fragment, and is marked in bold. Here we regard TFBSs or novel motifs that are both significant in O1 and P1 fragments as the over-represented TFBSs or over-represented novel motifs in boundaries of U-CGIs.

**Table 4:** The number of TFs with specific domains.

Domain	TFs in boundaries	TFs in control
Zinc finger C2H2-type domain	7	15
Others	3	74

We list the number of TFs with zinc finger C2H2-type domain and other domains in boundaries and control set.

We also looked for possible new motifs in the boundaries in the same way as mentioned in the last section, and got two over-represented novel motifs in the U-CGIs boundaries. They are listed at the bottom of Table 3, and their logos with the most similar known TFBSs can be found in the supplementary Table S4.

### Conservation of the putative TFBSs over-represented in U-CGIs and in the boundaries

We investigated the sequence conservation of the over-represented putative TFBSs across human, mouse and rat to examine how many of these sites are potentially functional (see Materials and Methods for details). We call a putative binding site significantly conserved if its *p*-value is less than 0.01. The proportion of significantly conserved binding sites among all the binding sites of each transcription factor is shown in supplementary Tables S5 and S6. One can see that the average proportion of significantly conserved binding sites of all over-represented putative TFBSs is 77.39% (with standard deviation 6.7%), which suggests that most of these TFBSs are under functional (negative) selection.

### Validation on two independent datasets

Yamada *et al* [28] profiled the methylation status of CGIs on human chromosome 21q from peripheral blood leukocytes. From their data, we got 104 U-CGIs and applied the same procedure to check the significance of the identified over-represented TFBSs based on the Rollins *et al*'s data (see results in Tables S7 and S8 in Supplementary Materials). It can be seen that 7 of the 10 TFBSs over-represented in U-CGIs and 10 of the 13 TFBSs over-represented in boundaries obtained in Rollins *et al*'s data are again significantly enriched in this dataset.

Recently, Schumacher *et al* [29] reported the profile of unmethylated sites on human chromosomes 21 and 22 in the brain tissue of eight adults. We extracted 61 U-CGIs from their unmethylated regions and applied our method (see results in Tables S9 and S10 in Supplementary Materials). It can be seen that 8 of the 10 over-represented TFBSs in U-CGIs and 9 of the 13 over-represented TFBSs in boundaries obtained in Rollins *et al*'s data are also significantly enriched.

From the results on these two independent datasets, we can see that most over-represented TFBSs identified in Rollins *et al*'s data are indeed enriched in all three independent data sets, suggesting that the existence of those putatively functional *cis*-acting elements in and around U-CGIs is robust and ubiquitous (largely independent of the examined tissue types).

### The influence of U-CGI localization preference

**Figure 3:**
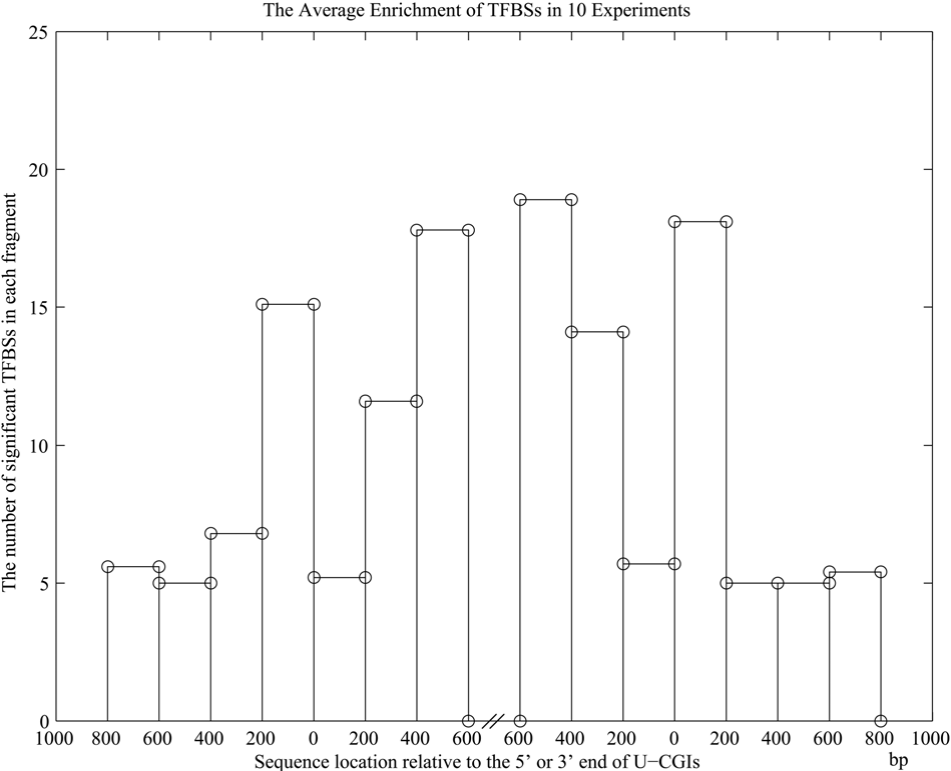
The number of significantly over-represented TFBSs in each fragment of promoter related CGIs and their flanking sequences. The *x*-axis indicates the location of fragment relative to the 5′ or 3′ end of CGIs. The *y*-axis is the average number of significant TFBSs in each fragment in the 10 experiments. One can see that there is no clear boundary in the flanking region of promoter related CGIs.

As is known, most of the U-CGIs are positioned at the 5′ end of human genes[1], which is also true for our dataset: 297 of the 304 U-CGIs are located in the promoter regions, while only 79 of the 210 M-CGIs are in promoter regions. It is necessary to check whether these over-represented *cis*-elements may simply reflect the difference between promoter-CGI (CGI located in promoter) and non-promoter-CGI (CGI not located in promoter) (see Materials and Methods for details). Results show that there is no clear boundary in the flanking region of the promoter related CGIs (Figure 3). There are still 3 over-represented TFBSs (V$HEN1_01, V$AP4 and V$HEB_Q6) in the promoter related CGIs in at least 5 of the experiments. However, when we changed the definition of over-represented TFBSs to be TFBSs that are significantly enriched in all the 6 U-CGI fragments, the first three TFBSs in Table 1 are still over-represented in the U-CGIs, while there is no over-represented TFBS in the 10 groups of promoter related CGIs. In summary, it is unlikely that the observed TFBSs over-represented in and around U-CGIs are merely caused by the difference between the CGIs in promoter and non-promoter, although this possibility could not be completely ruled out. As a matter of fact, positioning in the promoter region is one important feature of U-CGIs themselves. It is possible that more U-CGIs could be promoter related as many more promoters are still being discovered each day.

For our promoter related U-CGIs, one could see that the number of over-represented TFBSs in the 5′ and 3′ end of U-CGIs is nearly symmetrical (Figure 2). This is reasonable because we found that 72.4% (220 out of 304) of U-CGIs are located in bidirectional promoters (promoters shared by pairs of genes that flank them on the opposite strands). To study whether the symmetry also exists for U-CGIs located in the unidirectional promoters, we extracted the 71 U-CGIs located in unidirectional promoters on the positive strand and observed the similar symmetric distribution of the over-represented TFBSs (See Figure S3 in Supplementary Materials). Therefore, without loss of statistical power, we analyzed all the U-CGIs without subdividing the directions of their related promoters.

### Other chromatin marks mapped to the U-CGIs and their boundaries

**Figure 4:**
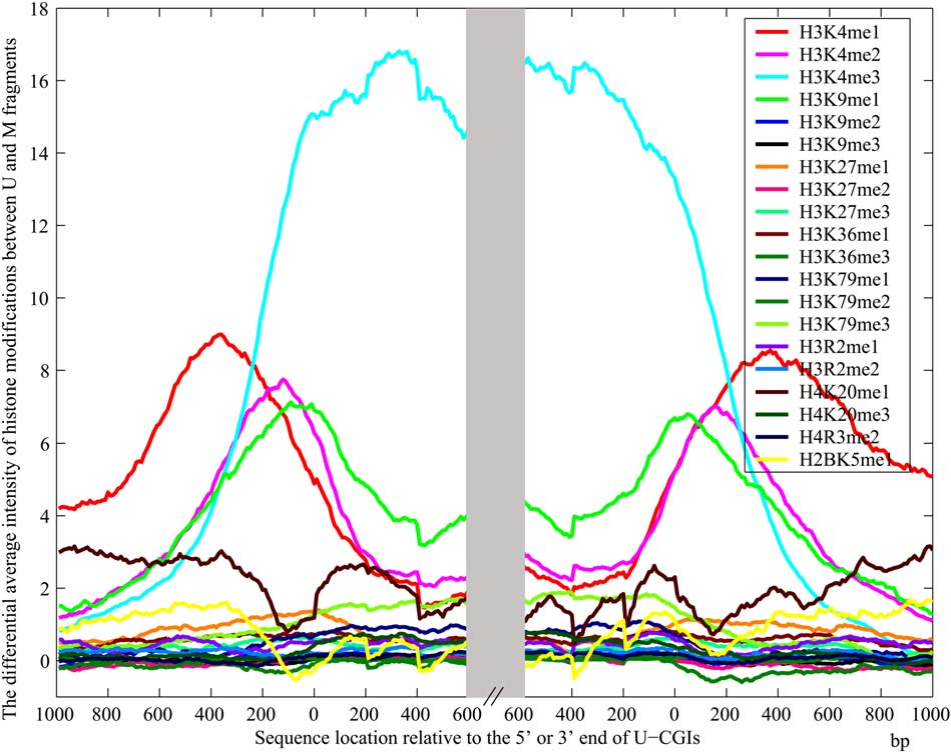
The average differences of histone modifications between U and M fragments. The *x*-axis indicates the location of fragment relative to the 5′ or 3′ end of CGIs. The *y*-axis is the differential average intensity in each fragment. One can see that H3K4me1, H3K4me2 and H3K9me1are enriched in boundary regions, and H3K4me3 is enriched both in the U-CGIs and their boundaries.

A large part of the TFs corresponding to the over-represented TFBSs act as activators, which could recruit co-activator complexes (such as chromatin-remodeling complexes, histone-modification enzymes) to modify the chromatin structure[30], [31]. It is interesting to study whether there are any correlated chromatin marks inside the U-CGIs and their boundaries. Currently there is only one high-resolution genome-scale profiling about histone modifications in human, which detected 20 histone methylations in T cells[32]. It has been reported that many tissues have similar DNA methylation landscapes, especially in CGIs[11], [12], [23]. Therefore, we investigated the intensities of the 20 histone modification marks in each fragment of the U- and M-CGIs and their flanking sequences. The average differences of the 20 histone marks between U-CGIs and M-CGIs are shown in Figure 4. One could see that H3K4me1, H3K4me2, H3K4me3 and H3K9me1, all of which were reported to be positively correlated with transcriptional levels[32]–[34], are enriched in boundary regions (H3K4me3 is also enriched inside U-CGIs). A more recent ChIP-seq study has further confirmed that CTCF marks boundaries of histone methylation domains [32]. The results imply that the U-CGIs and their boundaries may physically correspond to active chromatin domains and their barriers, many of the enriched ZF TFs may help recruiting chromatin-remodeling factors during the establishment and/or maintenance of the U-CGIs that are refractory to DNA methylation.

## Discussion

Of all the over-represented *cis*-acting elements in the boundaries of the U-CGIs, some of the corresponding TFs have been reported to prevent DNA methylation from spreading; while others still need to be further experimentally validated. In [14], [35], Brandeis *et al* and Mummaneni *et al* reported that Sp1 elements act as a barrier to impede the spreading of DNA methylation, later Sp1 and CTCF elements were observed to be boundary elements to maintain the methylation free state of *BRCA1* promoter in normal breast tissue [17]. CTCF is a transcriptional regulator with 11 zinc finger domains which exerts versatile function including repression, activation and chromatin insulation. Its methylation-dependent binding plays important roles in the regulation of imprinted genes (*H19/Igf2*) [36] and in X-chromosome inactivation [37]. Consistent with these observations, Sp1 and CTCF elements are both over-represented in our boundary regions. However the previously reported (ATAAA)_n_ boundary elements are not observed in our data, they are mostly found in repetitive heterochromatin regions that are under represented by the current human genome assembly. For the rest 11 TFBSs besides Sp1 and CTCF elements, there have been no reports about their potential roles in preventing methylation (notice that some of the TFBSs may be related, such as V$SP1, V$GC_01 and V$KROX_Q6 (reverse complement)). Furthermore, the Egr family corresponding to matrix V$KROX_Q6 has been recognized to be sensitive to methylation [38], and Sp3 corresponding to V$SP3 belongs to the same family with Sp1. Thus, it is possible that such TFBSs may have similar boundary functions. What is most surprising is that, 70% of the *trans*-acting factors corresponding to over-represented TFBSs in the boundaries of U-CGIs possess zinc finger (C2H2-type) DNA binding domains comparing to only 17% in control set (Table 4), and 74% of these putative binding sites are found in the evolutionarily conserved regions. Therefore, we suggest that these zinc finger DNA binding proteins are likely to play essential roles in blocking the spreading of DNA methylation and protecting the U-CGIs from being methylated.

Among the over-represented TFBSs within the U-CGIs, some of them are also present in the boundary sequences, e.g. V$SP1_01. HEN1 which is related to V$HEN1_01 and the latter is known to methylate *micro*RNAs in plants [39], and thus may in turn protect nearby sequences from methylation in plants since DNA methylation in plants could be directed by such *micro*RNAs [40]. But till now, there is neither a clear demonstration that RNA-directed *de novo* methylation exists in mammals [40], nor is there an evidence that HEN1 could methylate *micro*RNAs in mammals. Although many *cis*-acting elements in U-CGIs had been reported to be methylation sensitive, the understanding of their specific function is still controversial. Some reported that Sp1 elements in U-CGIs mask CGIs from *de novo* methylation directly [14] which would enhance the blocking function; others reported that Sp1 elements in U-CGIs are required for transcription and may only assist the unmethylation status indirectly [35]. We investigated the function annotations of TFs corresponding to the over-represented TFBSs in U-CGIs from Swiss-Prot. It turned out that 75% of them are annotated as activator, comparing to 50% in those over-represented in boundaries and 43% in the control TFs. Such TFs in U-CGIs should be closely related to activation of gene expression, which is consistent with earlier reports [4], [41]. Combining with the results that some over-represented TFBSs in U-CGIs are also present in boundary regions, we believe that these over-represented *cis*-acting elements in U-CGIs may have dual functions: they may help protecting U-CGIs against methylation, at the same time can also promote transcription [35].

It has been reported that H3K4 methylation coincides with CGIs to remarkable extent [42]. The targeting of the modification enzymes to the specific sites is largely dependent on gene-specific transcription factors [43]. The zinc finger proteins corresponding to the over-represented TFBSs might be co-regulators of the 4 enriched histone methylations. On the other hand, the histone modifications positively correlated with transcriptional levels have the potential to unfold chromatins, which can further strengthen the TFs' binding. The higher the probability of TFs' binding is, the stronger the methylation-protection function will be. DNA methylation and histone modifications are two important factors in the complex regulatory network modulating chromatin structure and genome function[44]. The correlation between the transcription factors, histone modification and CGI methylation observed in this study may reveal a clue on how the factors interact with each other in the complex network.

We also investigated whether there were some over-represented TFBSs in M-CGIs and their 400 bp flanking regions. Applying the same two-step hypothesis test on A2 vs. A1, F2 vs. F1, O2 vs. O1 and P2 vs. P1, we also got some over-represented TFBSs (see supplementary Tables S11 and S12). But there is no enriched chromatin marks in or around M-CGIs from Figure 4. Whether it is possible that the lacking of these TFBSs in the U-CGIs and in the boundaries may also contribute to methylation resistance can be an interesting topic for further investigations.

In mammalian genomes, methylation pattern is established by several steps during development [7]. Firstly, most of methyl groups are erased after fertilization [45]. Gametes will then undergo *de novo* methylation at about the time of implantation [46], and herefrom methylation pattern will be preserved by maintenance methylation during lifetime [47]. In the mouse, a hypothesis based on the *aprt* gene was proposed that methylation preferentially targets to specific sequences called methylation center in the *de novo* methylation stage [15], such as B1 repetitive elements [25]; then methylation will spread to their surrounding sequences till some *cis*-acting sequences block its spreading [15], and such barrier would explain the existence of the U-CGIs [40].

In the genome-wide U-CGIs obtained from human brain DNA, sequences adjacent to the unmethylation domains are occupied by *Alu*S and *Alu*Y families which are highly methylated [24]. In addition, we found that there are boundary regions of 400 bp both in the 5′ and 3′ end of U-CGIs over-represented with a group of zinc finger protein binding sites. Thus, we infer that the protection mechanism in the formation of U-CGIs based on mouse *aprt* gene is also applicable to human: *Alu* elements may function as the methylation centers [48]; when methylation moves forward their surrounding sequences, *cis*-acting elements bound with specific zinc finger proteins will block its spreading, and methylation pattern stabilizes when such counter-act reaches to a dynamical equilibrium. When some external condition changes, the dynamical equilibrium may be reestablished and the range of the methylation-resistant regions would move back and forth along the DNA sequences. For example, if some of the *cis*-acting elements are mutated (or methylation enzymes are mutated or their concentration levels are changed), the blocking capacity may be weakened or even dysfunctional, which would cause the U-CGIs to be gradually methylated. Similar observations have been reported in [14], [35]. Therefore, aberrant methylation of tumor-suppressor genes and progressive methylation in some tissue during aging may be explained by the weakening of the protection mechanism [49]. In our data, one U-CGI is in the promoter region of *MT1A* with *Alu* sequences in the 5′ flanking region. *MT1A* gene was reported to be methylation-free in normal tissue but hypermethylated to varying extent in some cancers [50], [51]. We speculate that a shifting of the dynamical equilibrium may contribute to the aberrant methylation.

## Materials and Methods

### Datasets

Our dataset is from Rollins *et al* [24], which detects the *in vivo* DNA methylation landscape of human brain. By digestion the sequences with McrBC (Rm^5^C-N_40–500_-Rm^5^C) and another five restriction endonucleases (REs): Tail (ACGT), BstUI (CGCG), HhaI (GCGC), HpaII (CCGG), and Acil (CCGC and GCGG), they identified 4240 methylation-resistant domains and 3518 methylation-prone domains respectively. According to the definition of CGI in UCSC Genome Browser: minimum length 200 bp, G+C content ≥50% and observed/expected CpG ratio ≥0.6 [10], we extracted 304 U-CGIs from methylation-resistant domains and 210 M-CGIs in methylation-prone domains as our background sequences. The median lengths of U-CGIs and M-CGIs are 886 bp and 275 bp respectively. To identify over-represented TFBSs, we used 146 non-redundant TFBSs corresponding to vertebrate TFs of TRANSFAC9.4 [27].

For validation, we obtained another two independent datasets. One is from Yamada *et al* [28]. They developed a simple HpaII-McrBC PCR method to discriminate the methylation status of CGIs on human chromosome 21q from peripheral blood leukocytes, and got 103 U-CGIs. As their definition of CGI (minimum length ≥400 bp, G+C content ≥50%, observed/expected CpG ratio ≥0.6) is different from ours, we applied our definition on their U-CGIs and obtained 104 U-CGIs. The other data is from Schumacher *et al* [29]. They applied tiling microarray technology to investigate the profiling of unmethylated sites on chromosome 21 and 22 in the brain tissue of eight adults respectively. Combining all the unmethylated regions of eight samples, we define such CGIs as U-CGIs if there are more than 100 bp overlap between the CGIs and these unmethylated regions. Finally, we extracted 61 U-CGIs from their data. The background sequences in the validation are also from M-CGIs as the methylation-prone data in the original datasets are too insufficient.

### Division strategy

In order to identify the sequence features in the U-CGIs, we divided the U-CGIs and the background CGIs into several 200 bp-long fragments, and all samples were aligned by the two ends of the CGIs (Figure 1). Such division strategy is based on the following reasons: firstly, the median length of the U-CGIs is much longer than median length of the M-CGIs, in order to avoid bias when comparing the foreground sequences (U) with the background sequences (M), we need to divide them into fragments with the same length; secondly, all of the CGIs are longer than 200 bp, setting the fragments to 200 bp would make full use of the samples; thirdly, we divided the U and the M-CGIs into 6 fragments and 2 fragments respectively because it would cover most of the sequences although some U-CGIs are longer than 1200 bp and some M-CGIs longer than 400 bp. Before this study, we knew that there should be some sequence features related to the formation of the U-CGIs in their flanking sequences [52], but we were unclear about how far the informative flanking sequences would extend. Therefore, we just arbitrarily extended 1200 bp around the U-CGIs at first, and also divided them into several 200 bp-long fragments. As there is no over-represented TFBS between the 800–1200 bp flanking regions, we only show the fragments in 800 bp flanking regions (Figure 1). It should be noted that some U-CGIs are less than 1200 bp and some M-CGIs are less than 400 bp. If any defined CGI fragment extends out of CGI, such fragment would be eliminated; likewise, if any fragment of flanking sequences surpasses the given domain, such fragment would be eliminated as well.

### Two-step hypothesis test

After the division processes, we applied MOTIFCLASS [26] to get the putative TFBSs in every fragment of both types of the CGIs and their flanking sequences respectively, and counted the number of samples with given TFBS. Then Fisher's exact test [53] was implemented in the U&M-CGIs and their flanking fragments by comparing (A1, B1, C1) with A2, (F1, E1, D1) with F2, J1 with J2, I1 with I2, H1 with H2, G1 with G2, K1 with K2, L1 with L2, M1 with M2, and N1 with N2. A TFBS with *p*-value less than Bonferroni-adjusted cutoff (0.01) is regarded as a statistically significant one. In order to filter the influence of the biased G+C content between U and M, we randomly shuffled fragments of both the U-CGIs and their boundary sequences for 1000 times, and compared the shuffled U fragments with M fragments to get the proportion of samples significantly enriched with given TFBS in the shuffled U fragments. Similar to a permutation test, we regard the ratio that the proportion with TFBS in shuffled U fragments is at least as extreme as the proportion in U fragments in 1000 times as the *p*-value in the shuffle test. And only the TFBS with *p*-value less than 0.01 would be regarded to have no biased G+C content influence. We define the two step processes as a two-step hypothesis test. Only the TFBS that is significant in both hypothesis tests is reported as the significant one.

### Novel motif identification

The processing described above only explores the known motifs (TFBSs). It is also meaningful to identify over-represented novel motifs in each of the fragment of the U-CGIs and the boundary sequences. Therefore, we applied DME-b [26] to hunt the novel motifs with width from 6 to 9 bp. For each motif width, we got the top 50 enriched motifs as candidate significant motifs. Considering that there would be some redundancies both between the novel motifs with different width themselves and between motifs and the over-represented TFBSs, we used the program Matcompare [54] to filter all the redundant novel motifs. The parameters of Matcompare are set as below: the greatest overhang when comparing motifs is 2, the Kullback-Leibler (K-L) divergence threshold is set to 1. If two motifs with different width are similar, the motif with larger width would be kept. In order to filter the influence of biased G+C content, we also implemented the two-step hypothesis test on the non-redundant motifs, and the *p*-value cutoff in the first step is also Bonferroni-adjusted (0.01).

### Promoter influence analysis

The promoter database is from CSHLmpd (http://rulai.cshl.edu/cshlmpd/). We defined the promoter to be the region between 1 kb upstream and 200 bp downstream of TSS (Transcription Starting Site). If a CGI overlaps with any promoter region, such CGI is regarded as promoter-CGI. In the process of identifying whether the over-represented TFBSs are only the difference between CGIs in promoter and non-promoter, the ideal method may be to implement the two-step hypothesis test on promoter-U-CGIs and promoter-M-CGIs. However, the strongly biased sample sizes between the promoter-U-CGIs and the promoter-M-CGIs as well as the small size of the promoter-M-CGIs compelled us to apply some other methods. We extracted all the CGIs in human genome and divided them into promoter-CGIs and non-promoter-CGIs. Then, by sampling data from the CGIs randomly, we constructed 10 groups of foreground sequences (promoter related CGIs) and background sequences (non-promoter related CGIs) with the same sample size and composition as U-CGIs and M-CGIs respectively, i.e. 304 foreground sequences (297 promoter-CGIs, 7 non-promoter-CGIs) and 210 background sequences (79 promoter-CGIs, 131 non-promoter-CGIs). The comparison process between foreground and background sequences is the same as U-CGIs vs. M-CGIs.

### Conservation analysis

We investigated the conservation information of the over-represented putative TFBSs among human, mouse and rat. With the multiple alignment results of human, mouse and rat on UCSC Genome Browser, we used the 100 bp upstream and downstream sequences of all the putative binding sites as the input sequences of PAML [55] to get their phylogenetic tree. Based on the phylogenetic tree, we applied MONKEY [56] to get the significance that each putative binding sites are more conserved among the three species than their flanking sequences. With the *p*-value cutoff of 0.01, we calculated the proportion of the significantly conserved binding sites among the over-represented putative binding sites.

### Chromatin marks information

Barski *et al* [32] generated a large scale high-resolution profiling of 20 histone methylations in human resting CD4^+^ T cells. We counted the number of each modification in a 200 bp window sliding with 10 bp offset in the U- and M-CGIs and their flanking sequences. The average intensity differences of the 20 chromatin marks in CGIs and 1000 bp flanking sequences between U and M are calculated at each location. The results are shown in Figure 4.

## Supporting Information

Figure S1 The number of over-represented TFBSs in each fragment of U-CGIs and their flanking sequences. The x-axis indicates the location of fragment relative to the 5′ or 3′ end of U-CGIs. Thus, fragments insides the two ‘0’s correspond to A1, B1, C1, D1, E1 and F1 of Figure 1 in the article, and fragments that are in the 800 bp upstream and downstream of U-CGIs represent J1, I1, H1, G1 and K1, L1, M1, N1 of Figure 1 in the article respectively. We applied the Bonferroni-adjusted p-value cutoff 0.05 here.
(6.81 MB TIF)

Figure S2 The number of over-represented TFBSs in each fragment of U-CGIs and their flanking sequences. The x-axis indicates the location of fragment relative to the 5′ or 3′ end of U-CGIs. Thus, fragments insides the two ‘0’s correspond to A1, B1, C1, D1, E1 and F1 of Figure 1 in the article, and fragments that are in the 800 bp upstream and downstream of U-CGIs represent J1, I1, H1, G1 and K1, L1, M1, N1 of Figure 1 in the article respectively. We applied the Bonferroni-adjusted p-value cutoff 0.001 here.
(6.89 MB TIF)

Figure S3 The number of over-represented TFBSs in each fragment of U-CGIs located in promoters of unidirectional genes and their flanking sequences. The x-axis indicates the location of fragment relative to the 5′ or 3′ end of U-CGIs. Thus, fragments insides the two ‘0’s correspond to A1, B1, C1, D1, E1 and F1 of Figure 1 in article, and fragments that are in the 800 bp upstream and downstream of U-CGIs represent J1, I1, H1, G1 and K1, L1, M1, N1 of Figure 1 in article respectively.
(6.84 MB TIF)

Table S1 The logos of over-represented TFBSs in U-CGIs.
(0.08 MB DOC)

Table S2 The logos of over-represented TFBSs in boundaries of U-CGIs.
(0.10 MB DOC)

Table S3 Motifs that are over-represented in U-CGIs. Also listed are the most similar TFBS to the motif, logo of the TFBS and their K-L divergence.
(0.04 MB DOC)

Table S4 Motifs that are over-represented in the boundary regions. Also listed are the most similar TFBS to the motif, logo of the TFBS and their K-L divergence.
(0.04 MB DOC)

Table S5 The proportion of the significantly conserved binding sites in all the over-represented putative binding sites of every TF in each U-CGI fragment.
(0.07 MB DOC)

Table S6 The proportion of the significantly conserved binding sites in all the over-represented putative binding sites of every TF in boundary sequences.
(0.08 MB DOC)

Table S7 Validation results in the U-CGIs of Yamada et al's data. The check mark in the table indicates that the TFBS is significantly enriched in the specific U-CGI fragment.
(0.04 MB DOC)

Table S8 Validation results in boundaries of U-CGIs of Yamada et al's data. The check mark in the table indicates that the TFBS is significantly enriched in the specific boundary region.
(0.04 MB DOC)

Table S9 Validation results in the U-CGIs of the Schumacher et al's data. The check mark in the table indicates that the TFBS is significantly enriched in the specific U-CGI fragment.
(0.04 MB DOC)

Table S10 Validation results in the boundaries of the U-CGIs of the Schumacher et al's data. The check mark in the table indicates that the TFBS is significantly enriched in the specific boundary region.
(0.04 MB DOC)

Table S11 Over-represented TFBSs in M-CGI fragments. The second column indicates whether the TFs corresponding to the over-represented TFBSs are expressed in human brain. The logos of the TFBSs are also given. The two numbers in the 4th-5th columns are the p-values in the two-step hypothesis test in the corresponding fragment (we use p1 and p2 to represent the upper and lower value respectively). Only the TFBS with p1 less than Bonferroni-adjusted p-value cutoff 0.01 and p2 less than 0.01 is regarded as a significant TFBS in the fragment, and is marked in bold. Here we regard TFBSs that are both significant in A2 and F2 fragments as the over-represented TFBSs in M-CGIs. The redundant TFBSs are eliminated according to MatCompare.
(0.12 MB DOC)

Table S12 Over-represented TFBSs in the 400 bp flanking regions of M-CGI. The second column indicates whether the TFs corresponding to the over-represented TFBSs are expressed in human brain. The logos of the TFBSs are also given. The two numbers in the 4th-5th columns are the p-values in the two-step hypothesis test in the corresponding fragment (we use p1 and p2 to represent the upper and lower value respectively). Only the TFBS with p1 less than Bonferroni-adjusted p-value cutoff 0.01 and p2 less than 0.01 is regarded as a significant TFBS in the fragment, and is marked in bold. Here we regard TFBSs that are both significant in O2 and P2 fragments as the over-represented TFBSs in flanking sequences of M-CGIs.
(0.05 MB DOC)
