# Supplementary figures and images for: Putative Zinc Finger Protein Binding Sites Are Over-Represented in the Boundaries of Methylation-Resistant CpG Islands in the Human Genome
Source: PLoS One. 2007 Nov 21;2(11):e1184. doi: 10.1371/journal.pone.0001184 (PMC2065907; doi:10.1371/journal.pone.0001184)

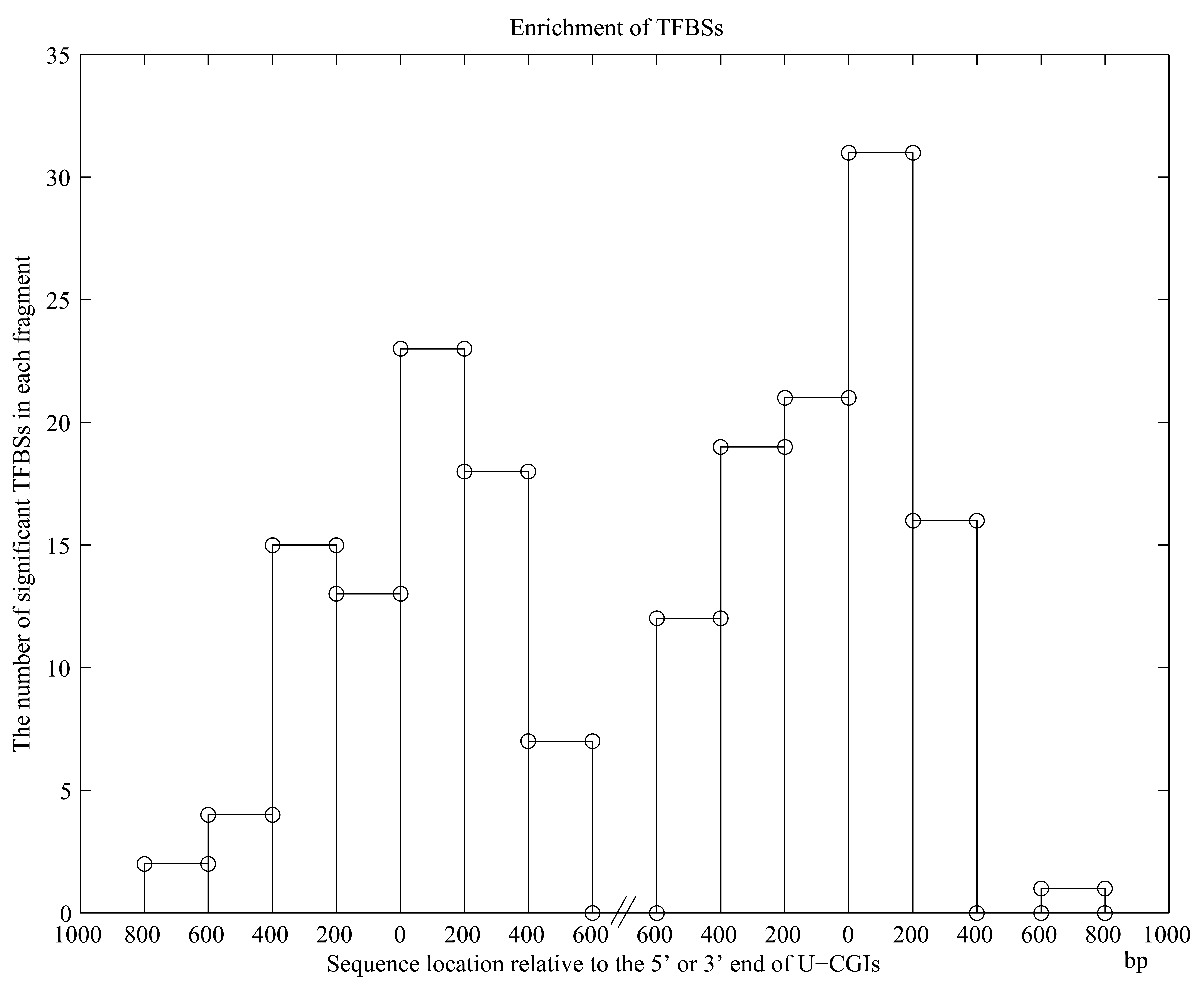

Supplement: Figure S1 — The number of over-represented TFBSs in each fragment of U-CGIs and their flanking sequences. The x-axis indicates the location of fragment relative to the 5′ or 3′ end of U-CGIs. Thus, fragments insides the two ‘0’s correspond to A1, B1, C1, D1, E1 and F1 of Figure 1 in the article, and fragments that are in the 800 bp upstream and downstream of U-CGIs represent J1, I1, H1, G1 and K1, L1, M1, N1 of Figure 1 in the article respectively. We applied the Bonferroni-adjusted p-value cutoff 0.05 here. (6.81 MB TIF) [file pone.0001184.s001.tif]

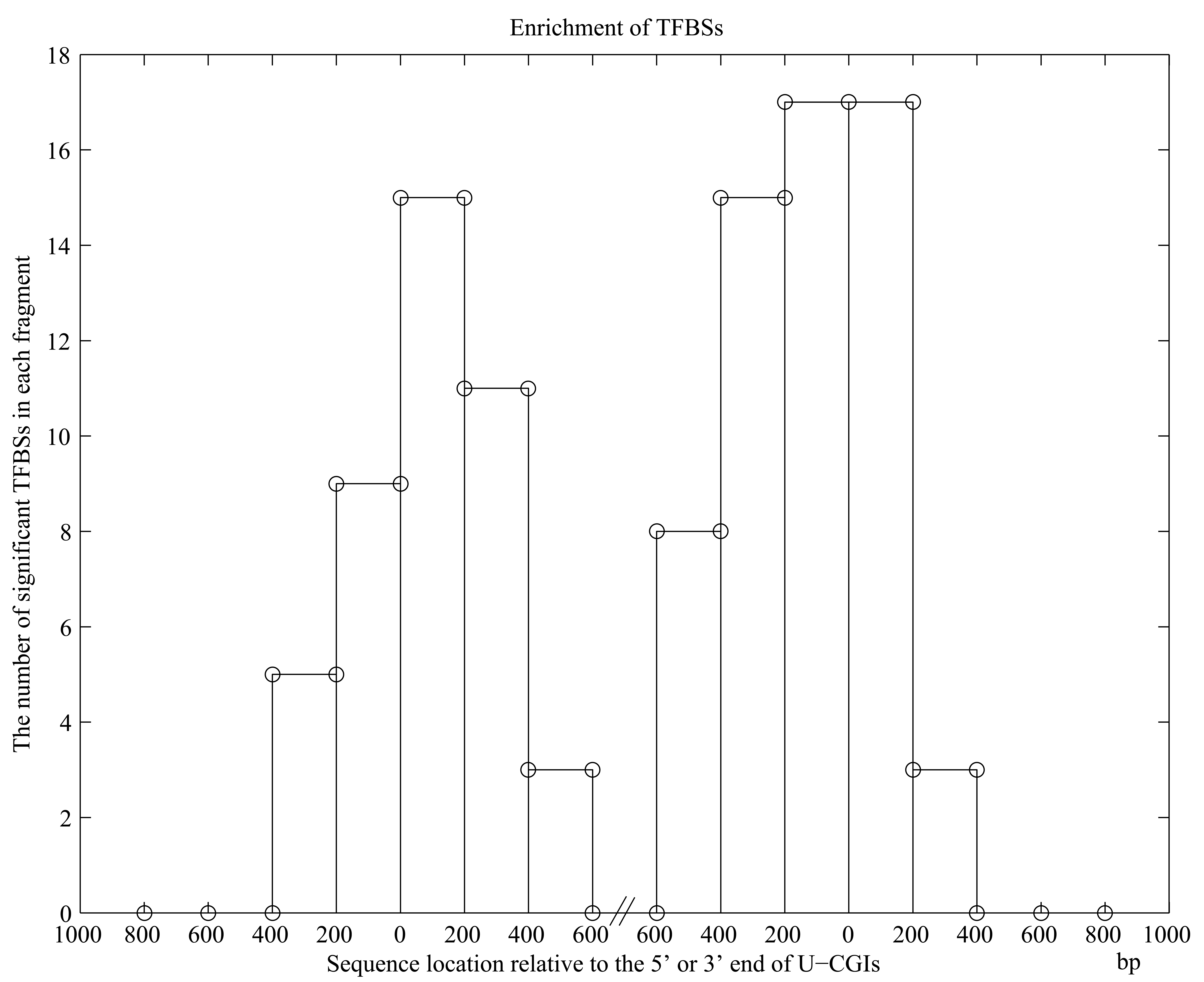

Supplement: Figure S2 — The number of over-represented TFBSs in each fragment of U-CGIs and their flanking sequences. The x-axis indicates the location of fragment relative to the 5′ or 3′ end of U-CGIs. Thus, fragments insides the two ‘0’s correspond to A1, B1, C1, D1, E1 and F1 of Figure 1 in the article, and fragments that are in the 800 bp upstream and downstream of U-CGIs represent J1, I1, H1, G1 and K1, L1, M1, N1 of Figure 1 in the article respectively. We applied the Bonferroni-adjusted p-value cutoff 0.001 here. (6.89 MB TIF) [file pone.0001184.s002.tif]

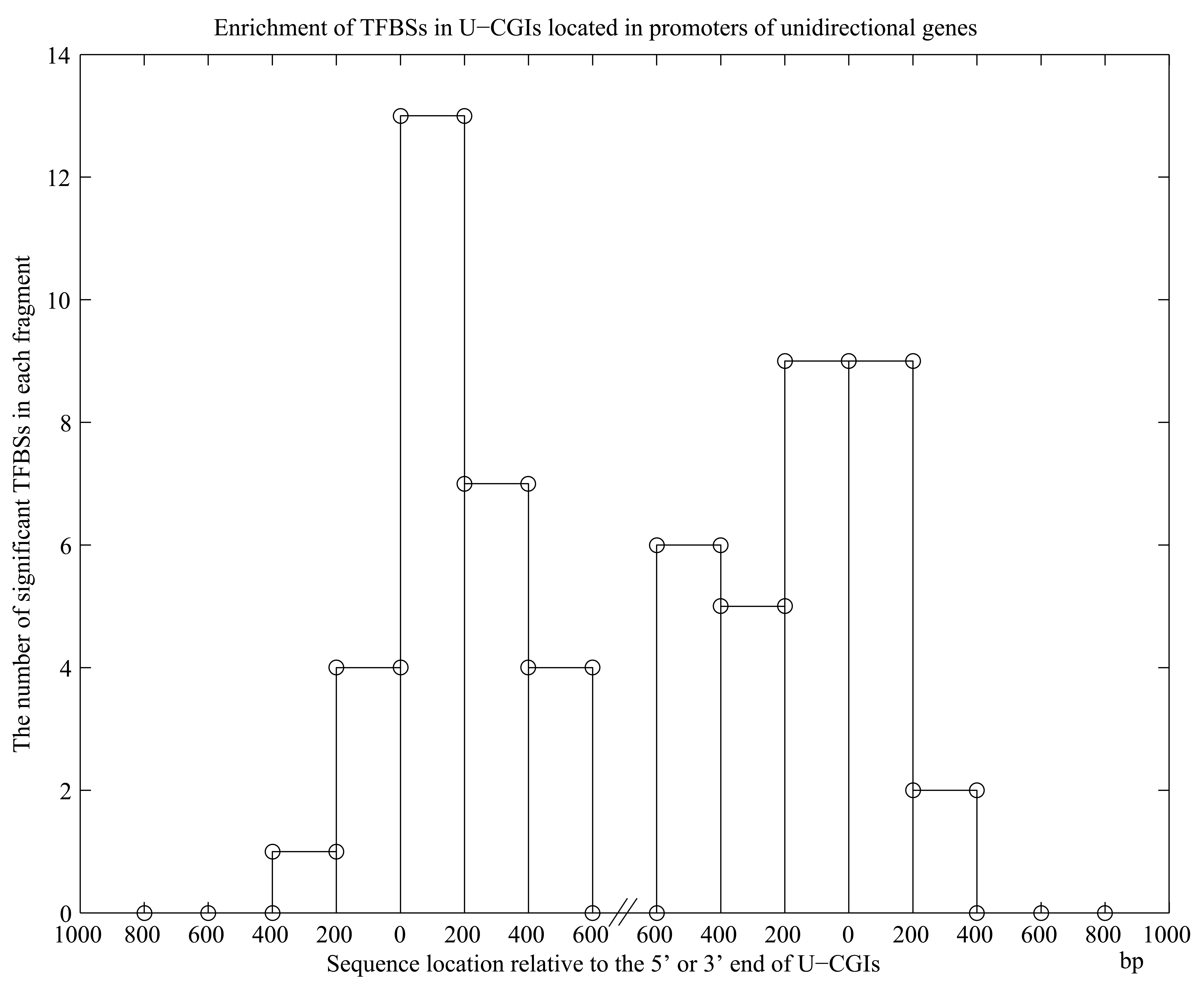

Supplement: Figure S3 — The number of over-represented TFBSs in each fragment of U-CGIs located in promoters of unidirectional genes and their flanking sequences. The x-axis indicates the location of fragment relative to the 5′ or 3′ end of U-CGIs. Thus, fragments insides the two ‘0’s correspond to A1, B1, C1, D1, E1 and F1 of Figure 1 in article, and fragments that are in the 800 bp upstream and downstream of U-CGIs represent J1, I1, H1, G1 and K1, L1, M1, N1 of Figure 1 in article respectively. (6.84 MB TIF) [file pone.0001184.s003.tif]
