# Supplementary material for: Putative Zinc Finger Protein Binding Sites Are Over-Represented in the Boundaries of Methylation-Resistant CpG Islands in the Human Genome
Source: PLoS One. 2007 Nov 21;2(11):e1184. doi: 10.1371/journal.pone.0001184 (PMC2065907; doi:10.1371/journal.pone.0001184)
Supplement: Table S1 — The logos of over-represented TFBSs in U-CGIs. (0.08 MB DOC) [file pone.0001184.s004.doc]

**Table S1.** The logos of over-represented TFBSs in U-CGIs.

| Over-represented TFBS | Logo |
| --- | --- |
| V$KROX_Q6 | 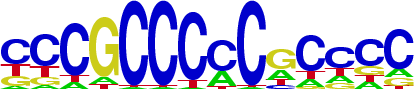 |
| V$SP1_01 | 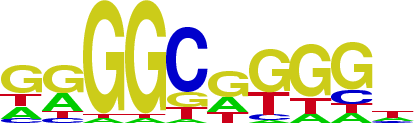 |
| V$HEN1_01 | 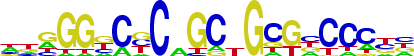 |
| V$CACBINDINGPROTEIN_Q6 | 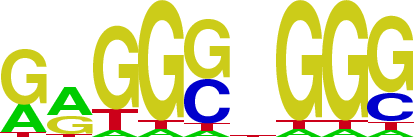 |
| V$PTF1BETA_Q6 | 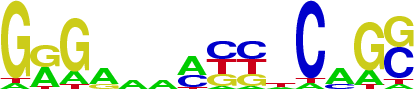 |
| V$AP4_01 | 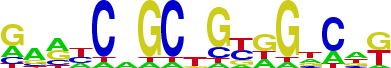 |
| V$DR1_Q3 | 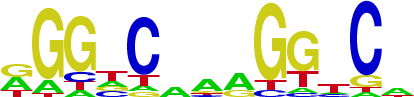 |
| V$ELK1_01 | 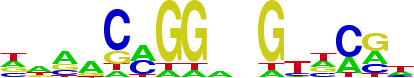 |
| V$PPAR_DR1_Q2 | 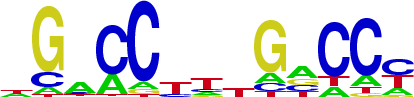 |
| V$HEB_Q6 | 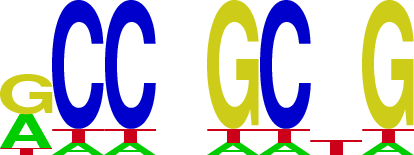 |
