# Supplementary material for: Putative Zinc Finger Protein Binding Sites Are Over-Represented in the Boundaries of Methylation-Resistant CpG Islands in the Human Genome
Source: PLoS One. 2007 Nov 21;2(11):e1184. doi: 10.1371/journal.pone.0001184 (PMC2065907; doi:10.1371/journal.pone.0001184)
Supplement: Table S2 — The logos of over-represented TFBSs in boundaries of U-CGIs. (0.10 MB DOC) [file pone.0001184.s005.doc]

**Table S2.** The logos of over-represented TFBSs in boundaries of U-CGIs.

| Over-represented TFBS | Logo |
| --- | --- |
| V$MAZR_01 | 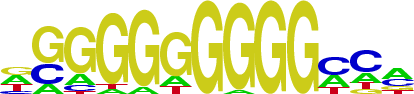 |
| V$CTCF | 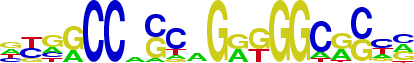 |
| V$ETF_Q6 | 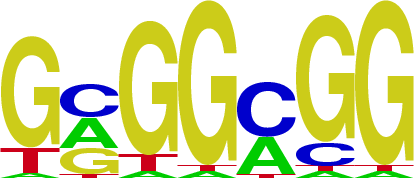 |
| V$AP2_Q3 | 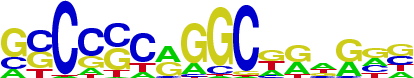 |
| V$SPZ1_01 | 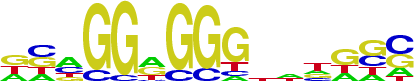 |
| V$KROX_Q6 | 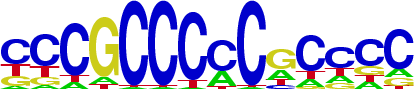 |
| V$CACBINDINGPROTEIN_Q6 | 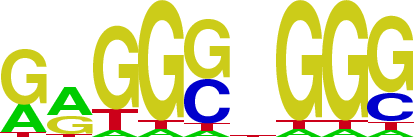 |
| V$NFKB_Q6 | 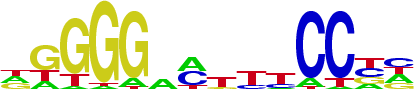 |
| V$TFIII_Q6 | 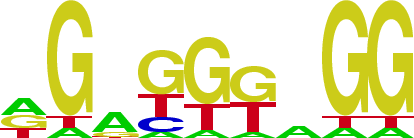 |
| V$MINI19_B | 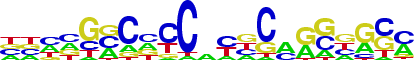 |
| V$GC_01 | 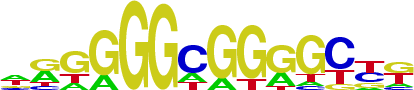 |
| V$SP3_Q3 | 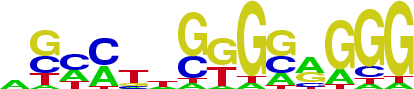 |
| V$SP1_01 | 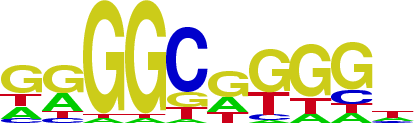 |
