# Supplementary material for: Putative Zinc Finger Protein Binding Sites Are Over-Represented in the Boundaries of Methylation-Resistant CpG Islands in the Human Genome
Source: PLoS One. 2007 Nov 21;2(11):e1184. doi: 10.1371/journal.pone.0001184 (PMC2065907; doi:10.1371/journal.pone.0001184)
Supplement: Table S3 — Motifs that are over-represented in U-CGIs. Also listed are the most similar TFBS to the motif, logo of the TFBS and their K-L divergence. (0.04 MB DOC) [file pone.0001184.s006.doc]

**Table S3.** Motifs that are over-represented in U-CGIs.

| DME-motif | Logo | Most similar TRANSFAC motif | | Divergence |
| --- | --- | --- | --- | --- |
| DME_CGI_1 | 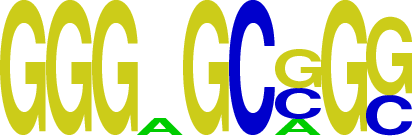 | V$KROX_Q6 | 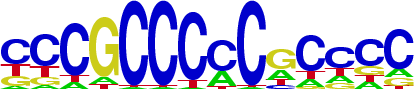 | 1.88386 |
| DME_CGI_2 | 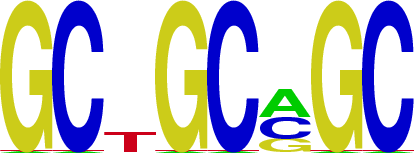 | V$AP4_01 | 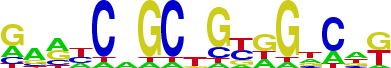 | 1.45048 |

Also listed are the most similar TFBS to the motif, logo of the TFBS and their K-L divergence.
