# Supplementary material for: Putative Zinc Finger Protein Binding Sites Are Over-Represented in the Boundaries of Methylation-Resistant CpG Islands in the Human Genome
Source: PLoS One. 2007 Nov 21;2(11):e1184. doi: 10.1371/journal.pone.0001184 (PMC2065907; doi:10.1371/journal.pone.0001184)
Supplement: Table S4 — Motifs that are over-represented in the boundary regions. Also listed are the most similar TFBS to the motif, logo of the TFBS and their K-L divergence. (0.04 MB DOC) [file pone.0001184.s007.doc]

**Table S4.** Motifs that are over-represented in the boundary regions.

| DME-motif | Logo | Most similar TRANSFAC motif | | Divergence |
| --- | --- | --- | --- | --- |
| DME_Boundary_1 | 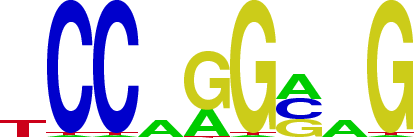 | V$MINI19_B | 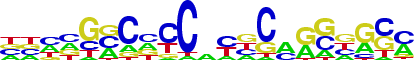 | 2.06672 |
| DME_Boundary_2 | 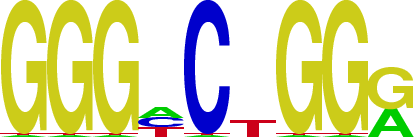 | V$SPZ1_01 | 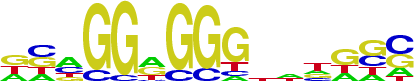 | 1.84366 |

Also listed are the most similar TFBS to the motif, logo of the TFBS and their K-L divergence.
