# Supplementary material for: Putative Zinc Finger Protein Binding Sites Are Over-Represented in the Boundaries of Methylation-Resistant CpG Islands in the Human Genome
Source: PLoS One. 2007 Nov 21;2(11):e1184. doi: 10.1371/journal.pone.0001184 (PMC2065907; doi:10.1371/journal.pone.0001184)
Supplement: Table S5 — The proportion of the significantly conserved binding sites in all the over-represented putative binding sites of every TF in each U-CGI fragment. (0.07 MB DOC) [file pone.0001184.s008.doc]

**Table S5.** The proportion of the significantly conserved binding sites in all the over-represented putative binding sites of every TF in each U-CGI fragment.

| Over-represented TFBS | Logo | A1 | B1 | C1 | D1 | E1 | F1 |
| --- | --- | --- | --- | --- | --- | --- | --- |
| V$KROX_Q6 | 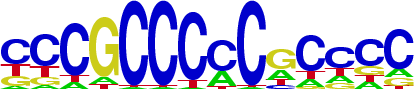 | 78.70% | 81.40% | 86.07% | 87.30% | 85.07% | 87.18% |
| V$SP1_01 | 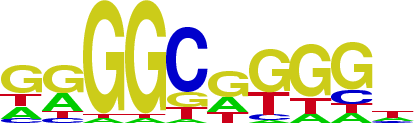 | 77.01% | 85.39% | 82.14% | 84.21% | 86.36% | 86.25% |
| V$HEN1_01 | 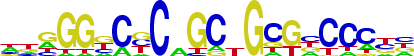 | 72.93% | 73.15% | 77.19% | 81.51% | 78.99% | 79.63% |
| V$AP4_01 | 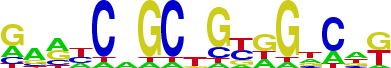 | 66.33% | 69.02% | - | - | 73.33% | 69.95% |
| V$DR1_Q3 | 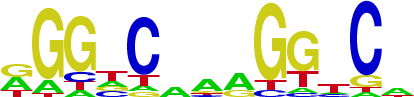 | 78.61% | 79.49% | - | - | 83.94% | 86.11% |
| V$ELK1_01 | 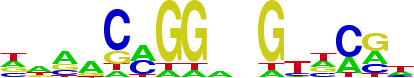 | 61.93% | 71.71% | - | 69.75% | - | 69.09% |
| V$PPAR_DR1_Q2 | 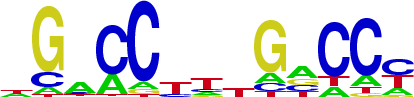 | 78.01% | 78.38% | - | - | 84.76% | 83.46% |
| V$HEB_Q6 | 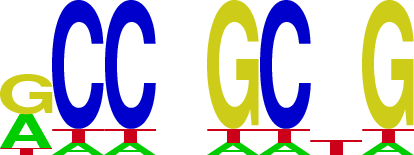 | 76.97% | 84.21% | - | 85.37% | - | 86.52% |
