# Supplementary material for: Putative Zinc Finger Protein Binding Sites Are Over-Represented in the Boundaries of Methylation-Resistant CpG Islands in the Human Genome
Source: PLoS One. 2007 Nov 21;2(11):e1184. doi: 10.1371/journal.pone.0001184 (PMC2065907; doi:10.1371/journal.pone.0001184)
Supplement: Table S6 — The proportion of the significantly conserved binding sites in all the over-represented putative binding sites of every TF in boundary sequences. (0.08 MB DOC) [file pone.0001184.s009.doc]

**Table S6.** The proportion of the significantly conserved binding sites in all the over-represented putative binding sites of every TF in boundary sequences.

| Over-represented TFBS | Logo | O1 | P1 |
| --- | --- | --- | --- |
| V$MAZR_01 | 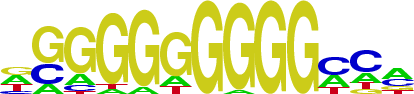 | 76.22% | 77.44% |
| V$CTCF | 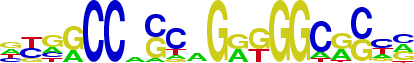 | 66.15% | 63.51% |
| V$AP2_Q3 | 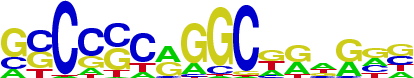 | 73.77% | 78.72% |
| V$SPZ1_01 | 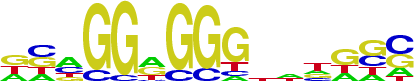 | 74.71% | 81.63% |
| V$KROX_Q6 | 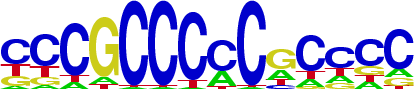 | 72.90% | 74.05% |
| V$NFKB_Q6 | 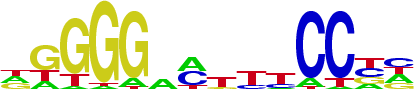 | 64.19% | 66.12% |
| V$TFIII_Q6 | 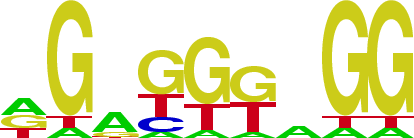 | 71.85% | 79.44% |
| V$GC_01 | 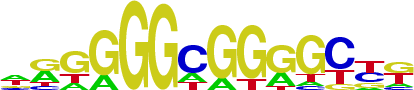 | 75.31% | 79.19% |
| V$SP3_Q3 | 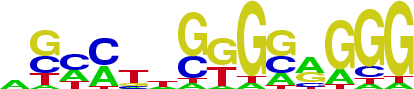 | 73.60% | 72.73% |
| V$SP1_01 | 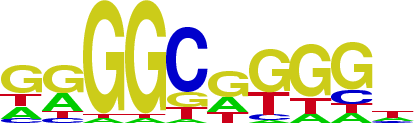 | 79.05% | 80.72% |
