# Supplementary material for: Putative Zinc Finger Protein Binding Sites Are Over-Represented in the Boundaries of Methylation-Resistant CpG Islands in the Human Genome
Source: PLoS One. 2007 Nov 21;2(11):e1184. doi: 10.1371/journal.pone.0001184 (PMC2065907; doi:10.1371/journal.pone.0001184)
Supplement: Table S7 — Validation results in the U-CGIs of Yamada et al's data. The check mark in the table indicates that the TFBS is significantly enriched in the specific U-CGI fragment. (0.04 MB DOC) [file pone.0001184.s010.doc]

**Table S7.** Validation results in the U-CGIs of Yamada *et al*’s data.

| Identified over-represented TFBSs from Rollins *et al*’s data | A1 | B1 | C1 | D1 | E1 | F1 |
| --- | --- | --- | --- | --- | --- | --- |
| V$KROX_Q6 | √ | √ |  |  |  |  |
| V$SP1_01 |  | √ | √ | √ |  |  |
| V$HEN1_01 |  |  |  |  |  |  |
| V$CACBINDINGPROTEIN_Q6 | √ |  | √ | √ |  |  |
| V$PTF1BETA_Q6 | √ |  |  |  |  |  |
| V$AP4_01 |  |  |  |  |  |  |
| V$DR1_Q3 |  | √ | √ |  | √ | √ |
| V$ELK1_01 | √ |  | √ |  |  | √ |
| V$PPAR_DR1_Q2 | √ |  | √ |  |  | √ |
| V$HEB_Q6 |  |  |  |  |  |  |

The check mark in the table indicates that the TFBS is significantly enriched in the specific U-CGI fragment.
