# Supplementary material for: Putative Zinc Finger Protein Binding Sites Are Over-Represented in the Boundaries of Methylation-Resistant CpG Islands in the Human Genome
Source: PLoS One. 2007 Nov 21;2(11):e1184. doi: 10.1371/journal.pone.0001184 (PMC2065907; doi:10.1371/journal.pone.0001184)
Supplement: Table S8 — Validation results in boundaries of U-CGIs of Yamada et al's data. The check mark in the table indicates that the TFBS is significantly enriched in the specific boundary region. (0.04 MB DOC) [file pone.0001184.s011.doc]

**Table S8.** Validation results in boundaries of U-CGIs of Yamada *et al*’s data.

| Identified over-represented TFBSs from Rollins *et al*’s data | O1 | P1 |
| --- | --- | --- |
| V$MAZR_01 | √ | √ |
| V$CTCF | √ |  |
| V$ETF_Q6 | √ | √ |
| V$AP2_Q3 | √ |  |
| V$SPZ1_01 | √ | √ |
| V$KROX_Q6 | √ | √ |
| V$CACBINDINGPROTEIN_Q6 | √ |  |
| V$NFKB_Q6 |  |  |
| V$TFIII_Q6 | √ |  |
| V$MINI19_B |  |  |
| V$GC_01 | √ |  |
| V$SP3_Q3 |  |  |
| V$SP1_01 | √ |  |

The check mark in the table indicates that the TFBS is significantly enriched in the specific boundary region.
