# Supplementary material for: Putative Zinc Finger Protein Binding Sites Are Over-Represented in the Boundaries of Methylation-Resistant CpG Islands in the Human Genome
Source: PLoS One. 2007 Nov 21;2(11):e1184. doi: 10.1371/journal.pone.0001184 (PMC2065907; doi:10.1371/journal.pone.0001184)
Supplement: Table S11 — Over-represented TFBSs in M-CGI fragments. The second column indicates whether the TFs corresponding to the over-represented TFBSs are expressed in human brain. The logos of the TFBSs are also given. The two numbers in the 4th-5th columns are the p-values in the two-step hypothesis test in the corresponding fragment (we use p1 and p2 to represent the upper and lower value respectively). Only the TFBS with p1 less than Bonferroni-adjusted p-value cutoff 0.01 and p2 less than 0.01 is regarded as a significant TFBS in the fragment, and is marked in bold. Here we regard TFBSs that are both significant in A2 and F2 fragments as the over-represented TFBSs in M-CGIs. The redundant TFBSs are eliminated according to MatCompare. (0.12 MB DOC) [file pone.0001184.s014.doc]

**Table S11.** Over-represented TFBSs in M-CGI fragments.

| TFBS | Expressed in human brain | Logo | A2 | F2 |
| --- | --- | --- | --- | --- |
| V$HNF1_Q6 | Y | 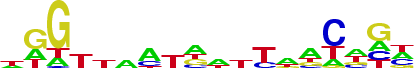 | **2.62e-45**  **0.000** | **3.12e-39**  **0.000** |
| V$CEBPGAMMA_Q6 | Y | 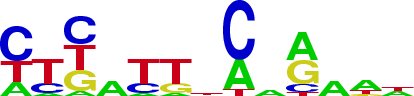 | **2.56e-39**  **0.000** | **1.13e-39**  **0.000** |
| V$IRF7_01 | Y | 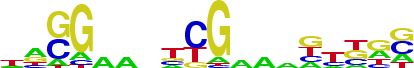 | **1.18e-37**  **0.000** | **3.37e-38**  **0.000** |
| V$CRX_Q4 | Y | 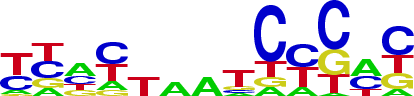 | **2.00e-32**  **0.000** | **5.40e-31**  **0.000** |
| V$AMEF2_Q6 | Y | 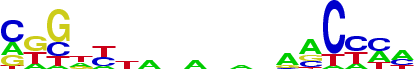 | **8.22e-31**  **0.000** | **7.53e-31**  **0.000** |
| V$CIZ_01 | Y | 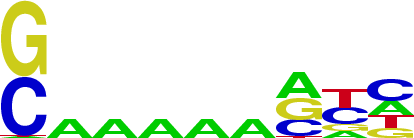 | **1.70e-29**  **0.000** | **2.42e-31**  **0.000** |
| V$HLF_01 | Y | 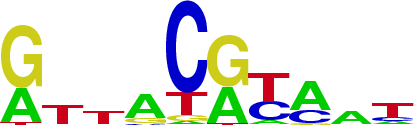 | **9.19e-29**  **0.000** | **1.50e-29**  **0.000** |
| V$LUN1_01 | Y | 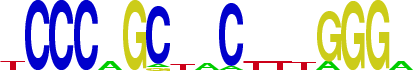 | **7.35e-28**  **0.000** | **1.72e-25**  **0.000** |
| V$RORA1_01 | Y | 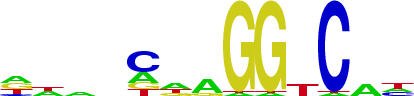 | **4.52e-22**  **0.000** | **1.32e-21**  **0.000** |
| V$ER_Q6 | Y | 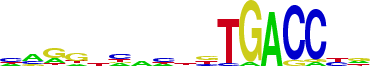 | **6.65e-19**  **0.000** | **1.16e-15**  **0.000** |
| V$SREBP1_01 | Y | 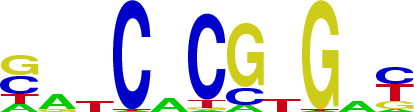 | **1.17e-15**  **0.000** | **2.71e-11**  **0.000** |
| V$ATF4_Q2 | Y | 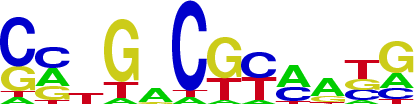 | **1.49e-13**  **0.000** | **4.56e-13**  **0.000** |
| V$GFI1_01 | Y | 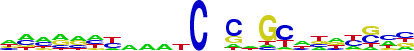 | **1.57e-13**  **0.000** | **8.10e-12**  **0.000** |
| V$CP2_02 | Y | 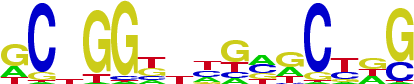 | **1.30e-09**  **0.000** | **3.38e-08**  **0.000** |
| V$ARP1_01 | Y | 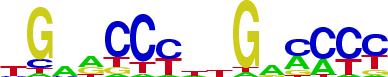 | **1.44e-08**  **0.000** | **4.98e-11**  **0.000** |
| V$ALX4_01 | Y | 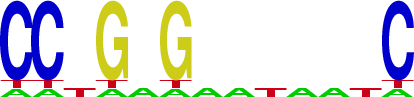 | **2.77e-08**  **0.001** | **2.37e-06**  **0.003** |
| V$TGIF_01 | Y | 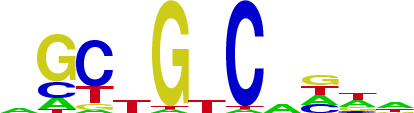 | **3.16e-08**  **0.003** | **3.61e-07**  **0.009** |
| V$PEBP_Q6 | Y | 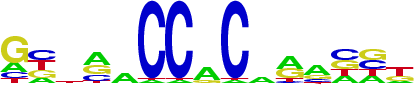 | **1.63e-06**  **0.000** | **2.37e-08**  **0.000** |

The second column indicates whether the TFs corresponding to the over-represented TFBSs are expressed in human brain. The logos of the TFBSs are also given. The two numbers in the 4th-5th columns are the *p*-values in the two-step hypothesis test in the corresponding fragment (we use$p_{1}$and$p_{2}$to represent the upper and lower value respectively). Only the TFBS with $p_{1}$ less than Bonferroni-adjusted *p*-value cutoff 0.01 and $p_{2}$ less than 0.01 is regarded as a significant TFBS in the fragment, and is marked in bold. Here we regard TFBSs that are both significant in A2 and F2 fragments as the over-represented TFBSs in M-CGIs. The redundant TFBSs are eliminated according to MatCompare.
