# Supplementary material for: Putative Zinc Finger Protein Binding Sites Are Over-Represented in the Boundaries of Methylation-Resistant CpG Islands in the Human Genome
Source: PLoS One. 2007 Nov 21;2(11):e1184. doi: 10.1371/journal.pone.0001184 (PMC2065907; doi:10.1371/journal.pone.0001184)
Supplement: Table S12 — Over-represented TFBSs in the 400 bp flanking regions of M-CGI. The second column indicates whether the TFs corresponding to the over-represented TFBSs are expressed in human brain. The logos of the TFBSs are also given. The two numbers in the 4th-5th columns are the p-values in the two-step hypothesis test in the corresponding fragment (we use p1 and p2 to represent the upper and lower value respectively). Only the TFBS with p1 less than Bonferroni-adjusted p-value cutoff 0.01 and p2 less than 0.01 is regarded as a significant TFBS in the fragment, and is marked in bold. Here we regard TFBSs that are both significant in O2 and P2 fragments as the over-represented TFBSs in flanking sequences of M-CGIs. (0.05 MB DOC) [file pone.0001184.s015.doc]

**Table S12.** Over-represented TFBSs in the 400bp flanking regions of M-CGI.

| TFBS | Expressed in human brain | Logo | O2 | P2 |
| --- | --- | --- | --- | --- |
| V$EVI1_04 | Y | 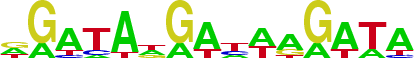 | **1.13e-11**  **0.000** | **4.01e-09**  **0.000** |
| V$HNF3_Q6 | Y | 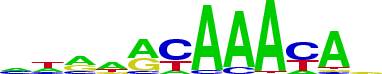 | **9.41e-10**  **0.000** | **3.74e-07**  **0.000** |
| V$FAC1_01 | Y | 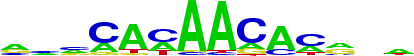 | **1.34e-09**  **0.000** | **4.77e-08**  **0.000** |
| V$HP1SITEFACTOR_Q6 | Y | 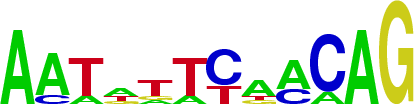 | **9.12e-06**  **0.000** | **1.20e-05**  **0.000** |
| V$MMEF2_Q6 | Y | 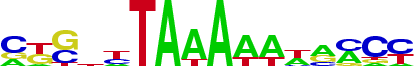 | **6.04e-06**  **0.000** | **1.94e-05**  **0.000** |

The second column indicates whether the TFs corresponding to the over-represented TFBSs are expressed in human brain. The logos of the TFBSs are also given. The two numbers in the 4th-5th columns are the *p*-values in the two-step hypothesis test in the corresponding fragment (we use$p_{1}$and$p_{2}$to represent the upper and lower value respectively). Only the TFBS with $p_{1}$ less than Bonferroni-adjusted *p*-value cutoff 0.01 and $p_{2}$ less than 0.01 is regarded as a significant TFBS in the fragment, and is marked in bold. Here we regard TFBSs that are both significant in O2 and P2 fragments as the over-represented TFBSs in flanking sequences of M-CGIs.
